# Evolution of high pathogenicity of H5 avian influenza virus: haemagglutinin cleavage site selection of reverse-genetics mutants during passage in chickens

**DOI:** 10.1038/s41598-018-29944-z

**Published:** 2018-08-01

**Authors:** Jasmina M. Luczo, Mary Tachedjian, Jennifer A. Harper, Jean S. Payne, Jeffrey M. Butler, Sandra I. Sapats, Suzanne L. Lowther, Wojtek P. Michalski, John Stambas, John Bingham

**Affiliations:** 1 Australian Animal Health Laboratory (AAHL), Commonwealth Scientific and Industrial Research Organisation (CSIRO), Geelong, Victoria Australia; 2 School of Medicine, Deakin University, Geelong, Victoria Australia; 3 Present Address: Center for Vaccines and Immunology, University of Georgia, Athens, Georgia United States of America

## Abstract

Low pathogenicity avian influenza viruses (LPAIVs) are generally asymptomatic in their natural avian hosts. LPAIVs can evolve into highly pathogenic forms, which can affect avian and human populations with devastating consequences. The switch to highly pathogenic avian influenza virus (HPAIV) from LPAIV precursors requires the acquisition of multiple basic amino acids in the haemagglutinin cleavage site (HACS) motif. Through reverse genetics of an H5N1 HPAIV, and experimental infection of chickens, we determined that viruses containing five or more basic amino acids in the HACS motif were preferentially selected over those with three to four basic amino acids, leading to rapid replacement with virus types containing extended HACS motifs. Conversely, viruses harbouring low pathogenicity motifs containing two basic amino acids did not readily evolve to extended forms, suggesting that a single insertion of a basic amino acid into the cleavage site motif of low-pathogenic viruses may lead to escalating selection for extended motifs. Our results may explain why mid-length forms are rarely detected in nature. The stability of the short motif suggests that pathogenicity switching may require specific conditions of intense selection pressure (such as with high host density) to boost selection of the initial mid-length HACS forms.

## Introduction

Since the emergence of the Eurasian-lineage H5N1 highly pathogenic avian influenza virus (HPAIV) in 1996, there has been continued spread and evolution of the virus. HPAIVs with Eurasian-lineage genes are now endemic in poultry in several countries including Vietnam, Indonesia, Bangladesh, India, China and Egypt^1,2^. Human infections with Eurasian H5N1 HPAIV were first detected in 1997, and of the 18 cases identified during the initial outbreak, six died from the infection^3^. Phylogenetic analysis traced the haemagglutinin gene lineage back to a progenitor virus of avian origin, A/goose/Guangdong/1/1996 (H5N1)^4^ and, to date, all human H5N1 HPAIV infections have originated from this Eurasian lineage^5,6^. Currently, human infections with H5N1 are associated with a 52% laboratory-confirmed case fatality rate^3,7^. HPAIV has the potential to cause widespread morbidity and mortality if it acquires the ability to sustain human-to-human transmission.

Infectivity of influenza viruses is dependent on numerous viral and host factors, such as expression of appropriate receptors in addition to receptor binding specificity^8^. However, proteolytic cleavage of the virus surface glycoprotein, the haemagglutinin (HA), plays a critical role^9^. The haemagglutinin cleavage site (HACS) motif of low pathogenicity avian influenza viruses (LPAIVs) typically contain one or two non-consecutive basic amino acid residues that are cleaved by trypsin and trypsin-like proteases with monobasic specificity^9–15^. Replication of LPAIVs is generally confined to trypsin-expressing epithelial cells^16^ of the respiratory and gastrointestinal tracts^17,18^. In contrast, the HACS motif of HPAIVs contains multiple basic amino acids^18,19^, facilitating cleavage by ubiquitously expressed proteases, most notably furin, with polybasic specificity^20,21^. This allows HPAIVs to replicate in multiple tissues, with a predilection in the chicken for replication in vascular endothelium^17,22–28^, which is considered to be a major factor responsible for high pathogenicity in this species.

Highly pathogenic avian influenza viruses evolve in high density chicken populations found in intensive commercial production systems^29–31^. For this reason, it is highly appropriate that cleavage site motif mutation and evolution of pathogenicity be studied in chickens. Analysis of naturally occurring H5N1 HACS motifs revealed a predilection for H5 HPAIVs to evolve to harbour seven basic residues in the HACS motif^19^, although viruses with fewer basic residues in the HACS motif can also be shown to exhibit similar high pathogenicity in chickens^32^. Importantly, removal of the HPAIV polybasic HACS (pHACS) motif by reverse genetics abrogates the ability of the virus to replicate in vascular endothelium and cause severe disease^27,33–36^. Several residues within the pHACS motif have been implicated as being critical to the pathogenicity of HPAIVs, in particular, the presence of basic residues at P1, P2, P4 and P6^35,37–40^. Despite this knowledge, the mechanism by which pHACS motifs evolve from shorter precursors is currently unknown.

The study described here investigated the process by which H5 avian influenza viruses evolve to acquire high pathogenicity. A panel of related Asian H5N1 HPAI viruses, derived from the parental A/Viet Nam/1203/2004 strain and differing only in their HACS motif, was generated by reverse genetics and evaluated for pathogenicity in chickens. Sequencing of rescued viruses isolated from chickens indicated fitness peaks at either a low pathogenic HACS motif (≤2 non-consecutive amino acids), or extended HACS motif (≥5 basic residues). We also tested the hypothesis that, given a suitable viral backbone, an H5N1 virus could rapidly evolve to higher pathogenicity if more than two basic residues were present within the HACS motif. The resultant viral pathotypes were assessed by multiple methods: (1) clinical pattern of infection in chickens, including time to death and clinical signs, and (2) pathogenesis of infection as determined by histopathological lesions and cell tropism of the virus. This knowledge contributes to the understanding of the molecular markers involved in HPAIV pathogenicity and the pathogenesis and evolution of viruses in avian hosts. It enhances our ability to predict the evolution of H5 high pathogenicity and to mitigate the potential for spillover events to humans.

## Results

A panel of viruses, derived from A/Viet Nam/1203/2004 (H5N1), were rescued by reverse genetics, differing only by their HACS motif sequence (Table 1). HA deduced amino acid sequences of viral stocks were verified by Sanger sequencing prior to infection studies. Engineered viruses were classified as harbouring the following HACS motifs: (1) short HACS motif (≤2 non-consecutive basic amino acid residues: rgLPAIV), (2) mid-length HACS motif (3–4 basic residues: Mutants 5, 6, and 7) and (3) extended HACS motif (≥5 basic residues: rgHPAIV, Mutants 1, 2, 3, 4, 8, and 9).

**Table 1:** Deduced amino acid sequences of engineered HACS motifs of reverse genetics viruses.

Reverse genetics virus	HACS motif ^a^												
	HA_1_												HA_2_
	P12	P11	P10	P9	P8	P7	P6	P5	P4	P3	P2	P1	P1’
rgHPAIV	N	S	P	Q	R	E	R	R	R	K	K	R	G
rgLPAIV	N	S	P	Q	R	E	—	—	—	—	T	R	G
Mutant 5	N	S	P	Q	R	E	—	—	—	—	K	R	G
Mutant 6	N	S	P	Q	R	—	—	—	—	K	T	R	G
Mutant 7	N	S	P	Q	—	—	—	—	R	K	K	R	G
Mutant 4	N	S	P	Q	R	E	—	—	R	K	K	R	G
Mutant 8	N	S	P	Q	—	—	—	R	R	K	K	R	G
Mutant 9	N	S	P	Q	R	E	—	R	R	K	K	R	G
Mutant 2	N	S	P	Q	R	E	R	R	R	K	T	R	G
Mutant 1	N	S	P	Q	R	E	R	R	Q	K	K	R	G
Mutant 3	N	V	P	Q	R	E	R	R	R	K	K	R	G

^1^Dash indicates deletion, underlined indicates mutation. Cleavage occurs C-terminally at the scissile bond between P1 (HA_1_) and P1’ (HA_2_). The HACS motif is flanked N-terminally by PQ, and C-terminally by G.

### *In silico* modelling of the H5 HA structure predicts efficient enzyme access and cleavage for extended HACS motifs

To predict the effect of introduced mutations on the structure of monomeric HA, protein homology modelling was performed using SWISS-MODEL^41–43^. The crystal structure of an H3 HA (PDB ID: 1HA0^44^) features a HACS motif in the precursor (uncleaved) state. For comparisons of rgHPAIV HA and engineered HA structures, with a particular focus on the HACS motif, this 1HA0 template adequately represents the native structure at this site, and therefore was selected as the template.

**Figure 1:**
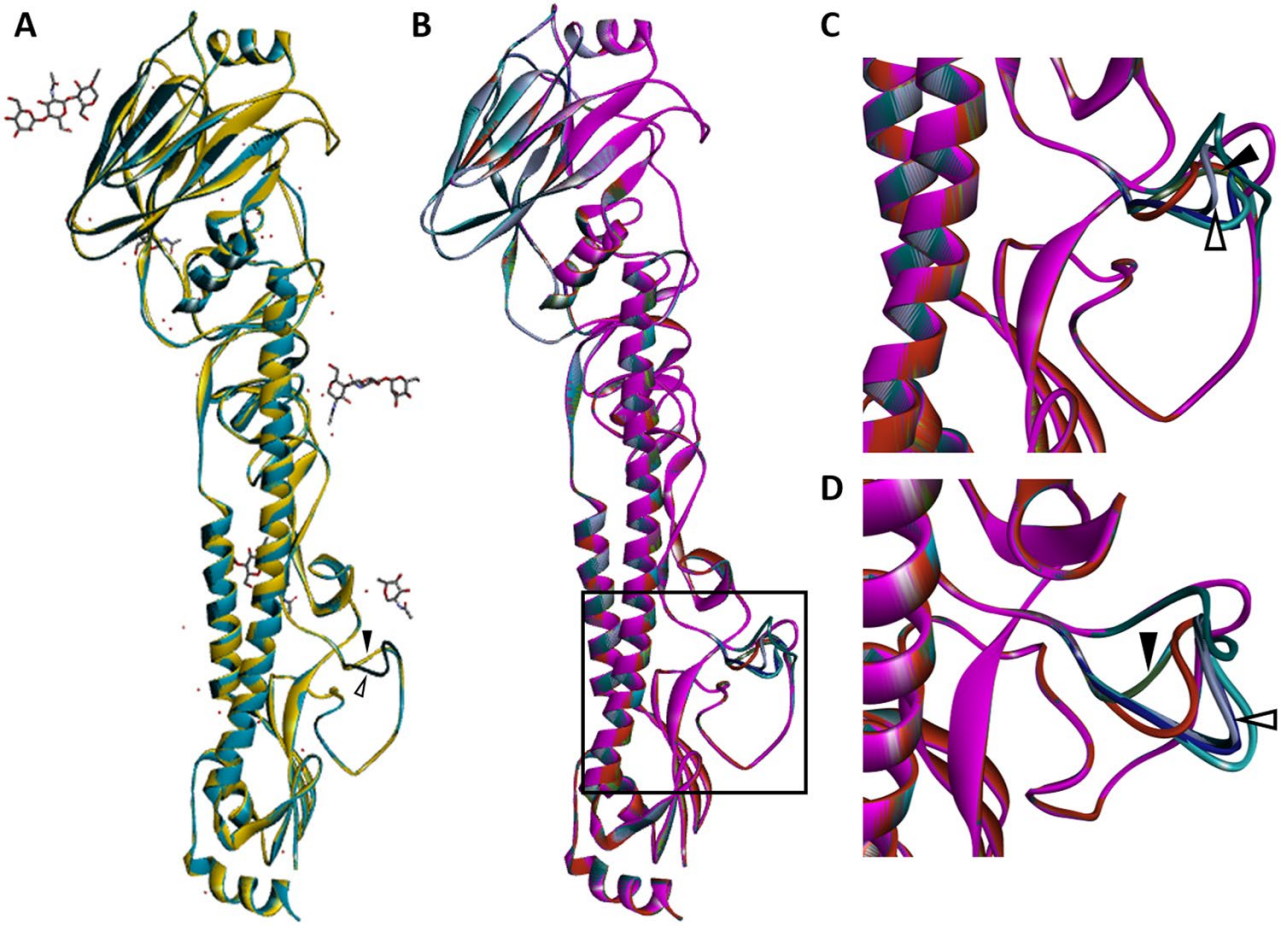
Homology model structure predictions of HA monomeric glycoproteins of rgHPAIV and engineered viruses. Protein tertiary structure prediction of engineered HA glycoprotein was performed by homology modelling (see text for details). The crystal structure of 1HA0, with intact loop structure, was used for template generation of predicted homology structure models. (**A**) Overlay of 1HA0, rgHPAIV and rgLPAIV (1HA0: gold; rgLPAIV: cyan; rgHPAIV: dark teal). Filled black arrowhead indicates 1HA0 and rgLPAIV loops; unfilled arrowhead indicates rgHPAIV loop. 1HA0 template carbohydrate moieties are indicated. (**B**) Overlay of predicted rgHPAIV and engineered HA monomers; loop structure that contains HACS motif is indicated in box (rgHPAIV, dark teal; rgLPAIV, cyan; M5, light green; M6, dark green; M7, purple; M4, light teal; M8, pink; M9, Red; M2, blue; M1, lilac; M3, dark orange). HA loop structures of mutants M5, M6 and M7 are congruent with the predicted loop structure of rgLPAIV; HA loop structures of M1, M2, M3, M4, M8 and M9 resemble the larger rgHPAIV loop structure, albeit, with variation. (**C**) Side view of predicted HA loop structures containing the HACS motif. D) Distal view of predicted HA loop structures containing the HACS motif. For (**C**) and (**D**) rgLPAIV and rgHPAIV predicted loop structures indicated by filled and unfilled arrowheads, respectively.

The predicted HA structures of rescued HPAIV virus (rgHPAIV) and engineered HA loops that harbour the HACS motif are solvent exposed and exhibit a degree of spatial variation (Fig. 1). Solvent exposure of the HA loops is anticipated to facilitate accessibility to, interaction with, and subsequent optimal cleavage by host cell proteases. The predicted HA loop structures of mutants M5, M6 and M7 are congruent with the predicted loop structure of rgLPAIV, although these viruses are predicted to be cleaved by furin (Supplementary Table S1). These mutant viruses with mid-length HACS motif may therefore exhibit sub-optimal interactions with furin, leading to decreased HA cleavage efficiency. The predicted HA loop structures of mutants M1, M2, M3, M4, M8 and M9 resemble the larger rgHPAIV solvent exposed loop, albeit with variation, which is likely to facilitate increasingly optimal accessibility and cleavage of the HACS motif by furin, suggesting that they are likely to exhibit high pathogenicity in chickens.

### H5 viruses expressing modified HACS motifs exhibit one of two pathotypes in chickens

**Figure 2:**
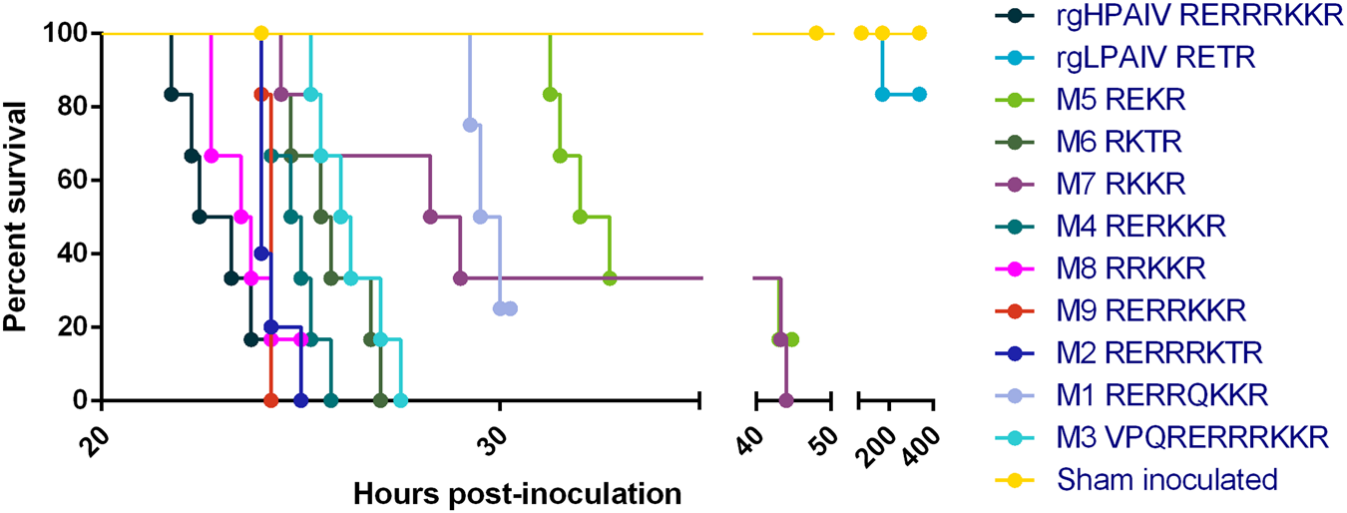
Survival curves of chickens following inoculation with reverse genetics generated viruses. Chickens were inoculated with ≥2 × 10^6^ TCID_50_ of infectious allantoic fluid by the ONO route and monitored for disease signs. Chickens were euthanased upon displaying neurological signs or manifestation of moderate HPAI-related disease signs. M1, M3-M7 exhibited significant prolonged survival times compared to rgHPAIV (M1 and M4, *P* < 0.01; M3, M5, M6 and M7, *P* < 0.001, sham inoculated, *P* = < 0.0001).

After generating reverse genetics viruses with various HACS motifs, pathogenicity in chickens was assessed. Inoculation of specific-pathogen free (SPF) chickens with rgHPAIV and mutants M1-M9 resulted in a peracute, fulminant disease consistent with HPAIV infection^18,27^. All chickens died within 48 hpi, although the majority died approximately 24 hpi. Chickens inoculated with mutants M1, M3–M7 exhibited significant prolonged survival times compared to rgHPAIV (M1 and M4, *P* < 0.01; M3, M5, M6 and M7, *P* < 0.001, sham inoculated, *P* < 0.0001), indicative of altered *in vivo* replication kinetics (Fig. 2, Table 2). Moreover, viral shedding from the oropharyngeal and cloacal routes also reflects the altered disease dynamics and delayed *in vivo* replication kinetics. While chickens inoculated with M1, M3-M7 had mean swab viral titres lower than rgHPAIV, chickens inoculated with M1 and M5 demonstrated noticeably reduced shedding from the oropharyngeal and cloacal routes at 24 hpi (Supplementary Fig. S1).

**Table 2:** Morbidity, mortality and survival times of SPF chickens inoculated with engineered reverse genetics viruses.

Reverse genetics virus	HACS motif	n	Morbidity (%)	Mortality (%)^a^	Survival time (hrs)			*P* value^b^
					Range	Mean	Median	
rgHPAIV	NSPQ_RERRRKKR/G	6	100	100	21.75–24.25	22.95	22.85	—
rgLPAIV	NSPQ_RETR/G	6	33	17	168.00			
Mutant 5	NSPQ_REKR/G	6	83	83	31.25–44.75	35.88	32.38***	0.0005
Mutant 6	NSPQ_RKTR/G	6	100	100	24.50–27.00	25.71	25.63***	0.0005
Mutant 7	NSPQ_RKKR/G	6	100	100	24.50–44.00	32.42	28.63***	0.0005
Mutant 4	NSPQ_RERKKR/G	6	100	100	24.00–25.75	24.83	24.88**	0.0033
Mutant 8	NSPQ_RRKKR/G	6	83	83	22.75–25.00	23.67	23.63	0.2354
Mutant 9	NSPQ_RERRKKR/G	6	100	100	24.00–24.25	24.21	24.25	0.0096
Mutant 2	NSPQ_RERRRKTR/G	5	100	100	24.00–25.00	24.25	24.00	0.0371
Mutant 1	NSPQ_RERRQKKR/G	4	75	75	29.25–30.25	29.75	29.75**	0.0038
Mutant 3	NVPQ_RERRRKKR/G	6	100	100	25.25–27.50	26.25	26.13***	0.0005

^a^Mortality refers to chickens requiring euthanasia at a pre-determined humane endpoint. Clinically healthy chickens were euthanased at the termination of the study (rgLPAIV, five of six chickens; M5, one of six chickens; M8, one of six chickens; M1, one of four chickens). ^b^Comparisons of survival curves with rgHPAIV were assessed using Log-rank (Mantel-Cox) test. Due to multiple comparisons, Bonferroni-corrected threshold adjusted to 0.004. **P < 0.01, ***P < 0.001.

Clinical disease signs included lack of eating/drinking, diarrhoea, ruffled feathers, listlessness, slow response time upon disturbance, isolation from the flock, sleepiness, drooping of the head, increased respiratory rate, huddling, severe depression and recumbency. Additionally, swelling of the eyelids and face, red conjunctivae and reddening of facial skin were commonly observed. Petechial haemorrhages were observed on facial skin, skeletal muscle and cardiac muscle. Transient neurological signs manifested in one chicken (M6, chicken #16). One chicken lacked clinical disease signs until convulsing prior to death (M9, chicken #26) and two chickens were found dead without observation of clinical signs (M6, chicken #18; M9, chicken #23). Three chickens (M5, chicken #7; M8, chicken #46; M1, chicken #10) were clinically healthy at the time of euthanasia.

In contrast to disease manifestation following inoculation with highly pathogenic viruses, inoculation of chickens with rgLPAIV resulted in no or mild clinical disease (Fig. 2, Table 2). Mild clinical disease signs included ruffled feathers and oedema of the eyelids (two of six chickens). One chicken (#93) exhibited ruffled feathers, drooping of the head and tail, hunched posture and neurological signs (mild torticollis, head twitching and ataxia). The latter was euthanased for welfare reasons.

### H5 highly pathogenic viruses exhibit high replicative capacity in multiple tissues

Our next objective was to compare replicative capacity of the viruses within tissues. Infectious virus was isolated from brain, spleen, lung and heart tissue excised from all HPAIV infected chickens 24–48 hpi (rgHPAIV, mutants M1-M9). Variation in tissue loads between challenge groups was not statistically significant. Generally, the greatest viral loads were detected in the spleen of all HPAIV inoculation groups, whereas the lowest viral loads were detected in brain tissue (Supplementary Fig. S2). The individual chickens that lacked clinical disease signs at the time of euthanasia (M5, #7; M8, #46; M1, #10) were positive for infectious virus in tissues, although these had the lowest virus titres in all within-group tissues analysed. Our results indicate that the replicative capacity in chicken tissues is similar across the HPAIV groups.

Following inoculation of chickens with rgLPAIV, infectious virus was isolated only from brain and lung tissue of chicken #93, which exhibited neurological sequelae and was humanely euthanased at 7 dpi. No virus was detected in tissues of remaining chickens, which were euthanased healthy at 14 dpi.

### Pathogenicity of HPAIVs in chickens correlates with endothelial tropism

Endothelial tropism is a feature of HPAI disease in chickens, which is thought to be a major determinant of high pathogenicity in this species^17,22–28^. To support the view that the pathogenicity of HPAIVs is based on endothelial tropism in our treatment groups, we assessed vascular tropism using immunohistochemistry.

**Figure 3:**
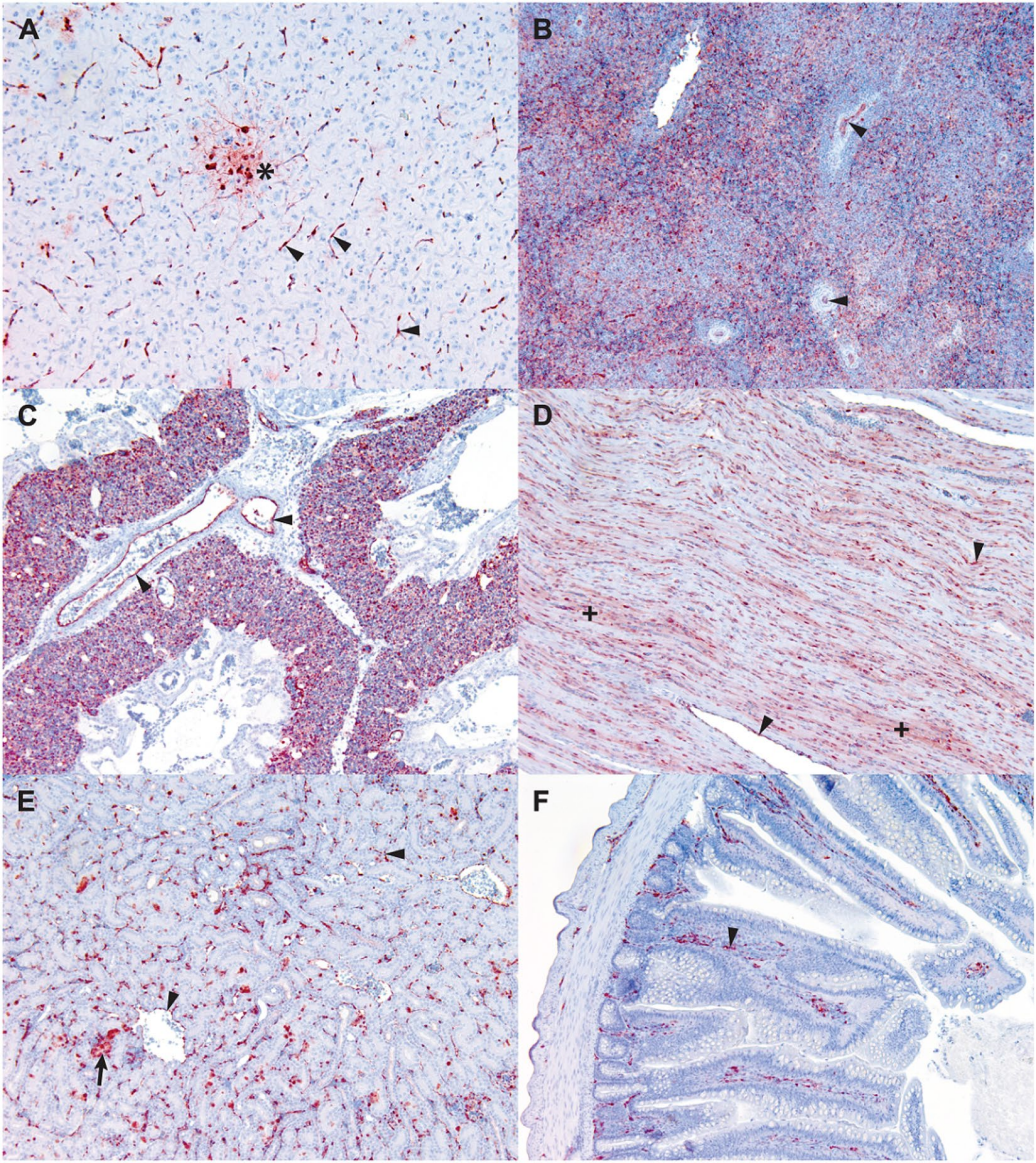
Viral antigen in rgHPAIV infected tissues. *Influenzavirus A* nucleoprotein antigen (brown) detected by immunohistochemistry in various tissues. Vascular endothelium of capillaries and larger blood vessels is the dominant cell type supporting viral antigen in all tissues (arrowheads). (**A**) Brain, showing antigen in capillary endothelium and also in a focus of neurons and astrocytes (*). (**B**) Spleen, showing antigen in endothelium of arterioles and also associated with non-lymphocytic cells of the splenic parenchyma. (**C**) Lung, showing antigen in vascular endothelium; the main antigen staining is in the rich capillary network of the interstitium. (**D**) Heart, showing antigen in endothelium and in cardiomyocytes, where it is seen as a brown hue within the muscle fibres (+). (**E**) Kidney: antigen is mainly in the interstitial capillaries, but also in occasional tubular epithelial cells (arrow). (**F**) Jejunum, where antigen is present in capillaries within the laminar propria.

Nucleoprotein viral antigen was observed in all organ systems examined (Supplementary Table S2). Antigen derived from highly pathogenic viruses (rgHPAIV, mutants M1-M9) was observed in multiple cell types, with vascular endothelium the predominant cell type supporting virus replication (Fig. 3). This was the case in all chickens where highly pathogenic disease manifested. Viral antigen was also common or abundant in neurons and astrocytes (Fig. 3A), in mononuclear phagocytic tissue such as spleen (Fig. 3B), in lung parenchyma (Fig. 3C), in cardiomyocytes (Fig. 3D) and occasional kidney tubular epithelial cells (Fig. 3E). Antigen was generally rare in epithelium, although it was dense within the sub-epithelial vascular network, such as the lamina propria of the intestine (Fig. 3F).

In contrast to the widespread tissue tropism exhibited by highly pathogenic viruses, viral antigen was not observed in endothelium of rgLPAIV infected tissues. In chicken #93, which exhibited neurological signs, there was diffuse focal viral antigen within the neurons of the brain, occasional foci of antigen in the epidermis of the comb (Supplementary Fig. S3) and rare antigen in the lungs, the cardiomyocytes and within interstitial inflammatory lesions of the kidneys.

### HPAIVs with extended pHACS sequences are rapidly selected *in vivo*

The results heretofore describe features of the infection and disease caused by the different reverse genetics viruses and have indicated that the disease outcome of all, except rgLPAIV, is consistent with highly pathogenic disease. Given that advanced infection is caused not by inoculated virions, but by their progeny following replication and selection, we sought to determine the viral HACS motif sequence of the viral population at the time of disease. Nucleic acid extracted from infected brain and/or lung tissue, collected at times described above, was amplified by PCR and sequenced by Sanger sequencing to determine if additional modifications had occurred within the HACS motif following *in vivo* passage.

Viral HA nucleic acid sequences were undetectable in the lung and brain tissue of rgLPAIV inoculated chickens, with the exception of chicken #93, which had exhibited neurological signs (the HA sequence isolated from lung and brain tissue remained unchanged). The pHACS motif of rgHPAIV and other highly pathogenic viruses (mutants M2, M3, M4, M8, and M9) isolated from lung and/or brain tissue also remained stable following *in vivo* passage. Mid-length pHACS motifs (mutants M5, M6 and M7) were unstable and incorporated additional basic amino acids following *in vivo* passage (Table 3). There was no variation in nucleic acid sequences isolated from lung or brain tissue, suggesting that selection of viruses with extended motifs is not tissue specific.

**Table 3:** Nucleotide and deduced amino acid sequence of HACS motif of viral inocula and following viral passage in chickens.

Virus	*n* ^a^	Parental HACS motif	HACS motif post-passage^b^
rgHPAIV	6	NSPQRERRRKKR/G aat agc cct caa aga gag aga aga aga aaa aag aga/gga	NC
rgLPAIV	6	NSPQRETR/G aat agc cct caa aga gag ac gaga/gga	NC
Mutant 5	6	NSPQREKR/G aat agc cct caa aga gag aag aga/gga	NSLRREKR/G (n = 5) aat agc ctt cga aga gag aag aga/gga NSHQREKR/G (n = 1) aat agc cat caa aga gag aa gaga/gga
Mutant 6	6	NSPQRKTR/G aat agc cct caa aga aaa acg aga/gga	NSPQRK*RK*TR/G (n = 6) aat agc cct caa aga aaa a*ga aaa a*cg aga/gga
Mutant 7	6	NSPQRKKR/G aat agc cct caa aga aaa aag aga/gga	NSPQR*R*KKR/G (n = 6) aat agc cct caa aga a*ga a*aa aag aga/gga
Mutant 4	6	NSPQRERKKR/G aat agc cct caa aga gag aga aaa aa gaga/gga	NC
Mutant 8	6	NSPQRRKKR/G aat agc cct caa aga aga aaa aag aga/gga	NC
Mutant 9	6	NSPQRERRKKR/G aat agc cct caa aga gag aga aga aaa aag aga/gga	NC
Mutant 2	5	NSPQRERRRKTR/G aat agc cct caa aga gag aga aga aga aaa acg aga/gga	NC
Mutant 1	4	NSPQRERRQKKR/G aat agc cct caa aga gag aga aga caa aaa aag aga/gga	NSPQRERRRKKR/G (n = 1) aat agc cct caa aga gag aga aga cga aaa aag aga/gga NSPQRERRLKKR/G (n = 2) aat agc cct caa aga gag aga aga cta aaa aag aga/gga NSPQRKRRQKKR/G (n = 1) aat agc cct caa aga aag aga aga caa aaa aag aga/gga
Mutant 3	6	NVPQRERRRKKR/G aat gtc cct caa aga gag aga aga aga aaa aag aga/gga	NC

^a^*n*: number of chickens.

^b^Italicised font indicates insertions. Underlined indicates mutation(s). NC: no change. The nucleic acid sequence isolated post-passage was taken from lung and/or brain tissue.

HPAIVs with mid-length HACS motifs are predicted to have a HA loop structure congruent with the loop structure of rgLPAIV (Fig. 1), potentially influencing the fitness of highly pathogenic viruses, as a result of suboptimal interactions with furin’s active site. Sequencing of M6 and M7 after *in vivo* passage revealed insertions of basic amino acids in the HACS motif (Table 3), which would serve to increase the stability of the interaction with furin. The HACS motif of mutant M1, that initially harboured glutamine at P4, mutated in three of four chickens, to incorporate either a leucine, to revert to an arginine, or to incorporate a compensatory lysine at P7 (Table 3). The compensatory mutation at P7 (lysine), may serve to reinstate a substrate with sufficient basic amino acids to overcome the suboptimal amino acid at P4. Furthermore, it has been reported that aliphatic residues at P4 (such as leucine) are tolerated by furin^45^, likely facilitating the substitution mutation from glutamine to leucine identified at this position. Sequencing of viruses isolated from M5 inoculated chickens revealed substitutions at P6 to either basic (histidine) or aliphatic (leucine) amino acids (Table 3). Despite delayed infection kinetics *in vivo*, this did not alter HPAI disease outcome. The HACS motif of all remaining highly pathogenic viruses (rgHPAIV, M2, M3, M4, M8, and M9) remained stable following *in vivo* passage, suggesting that the HACS motif could sufficiently interact with the activating protease, as predicted by the HA loop structures (Fig. 1).

In summary, our HACS motif sequence analysis suggested that viruses harbouring two non-consecutive basic residues (rgLPAIV) within a HACS motif were stable upon *in vivo* passage, as were pHACS motifs harbouring five or more basic residues. Viruses harbouring HACS motifs that contained three to four basic residues (mutants M5, M6 and M7) or non-basic residues at key positions, rapidly changed to be superseded by viral populations incorporating additional basic residues in the HACS motif.

### Initial replication in respiratory epithelium is not an absolute requirement for H5 HACS motif expansion

Initial studies revealed that mid-length pHACS motifs were displaced by extended motifs during replication in chickens (Table 3). To examine this process in more detail, we compared shifts in viral populations, focusing on the HACS motif cDNA sequence, between inocula and following disease onset, using next generation sequencing (NGS). We also compared how different routes of inoculation could affect the composition of the pHACS motif sequence. To this end, different groups of SPF chickens were inoculated with the same stock of M6 by either the oronasal-ocular (ONO) or intravenous (IV) route (*n* = 6 chickens each). This HPAIV challenge virus was selected for this study as it harboured a mid-length pHACS motif present in naturally occurring viruses^19,46,47^, is a possible transition species during pathotype conversion, and consistently incorporated additional basic residues into the HACS motif. In our hypothesis, we expected that the dominant (mid-length) HACS sequence of the inoculum would be given a more favourable opportunity to replicate sustainably and be detected if inoculated IV, before virions with extended-length sequences became the dominant type.

**Figure 4:**
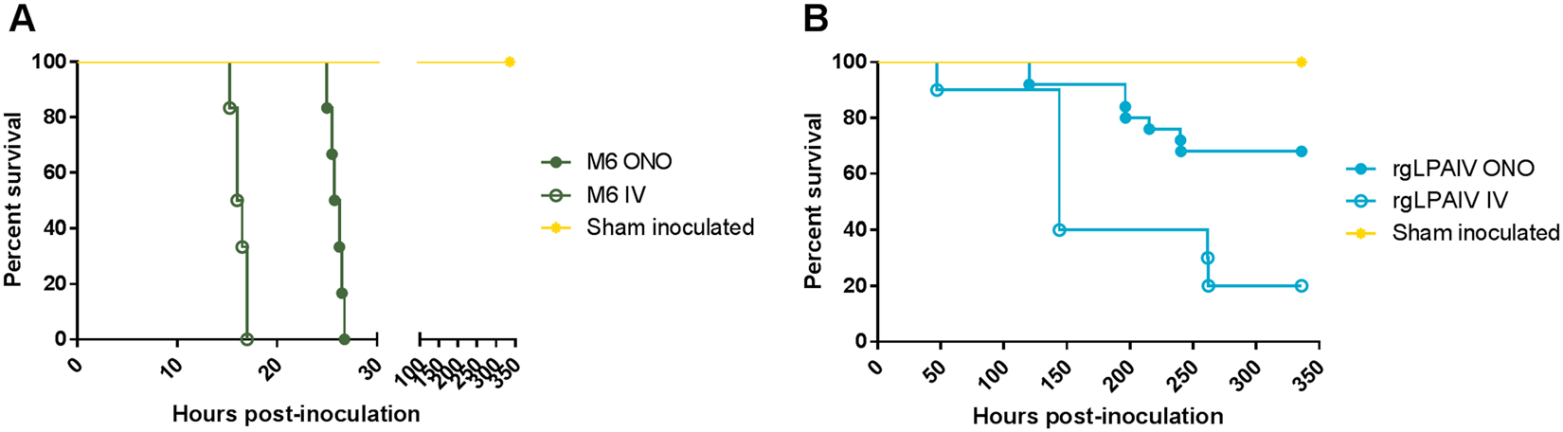
Survival curves of chickens following challenge with M6 or rgLPAIV (second challenge trials). Chickens were inoculated with ≥2 × 10^6^ TCID_50_ mutant M6 (**A**) or rgLPAIV (**B**) by the oronasal-ocular (filled circles) or intravenous (unfilled circles) route, and monitored for disease signs. Chickens were euthanased upon displaying neurological signs or manifestation of moderate HPAI-related disease signs.

Chickens inoculated with M6 by the ONO (*n* = 6) and IV (*n* = 6) route displayed peracute, fulminant clinical signs typical of HPAI infection: infrequent eating and drinking, ruffled feathers, diarrhoea, listlessness, slow response time upon disturbance, isolation from the flock, sleepiness, huddling, drooping of the head, severe depression and recumbency. Median survival times of chickens inoculated by the ONO and IV routes were 26.0 and 16.3 hrs, respectively (Fig. 4), a difference that was statistically significant (Log-Rank (Mantel-Cox) test, *P* = 0.0007). Viral antigen was observed in all organ systems examined (Supplementary Table S3). Histopathological signs were consistent with HPAI disease and immunohistochemistry confirmed the presence of viral antigen in endothelium for all chickens in both M6 inoculated groups.

Sequencing of viral nucleic acid extracted directly from infected brain tissue of both M6 challenge groups revealed a duplication event within the HACS motif, consisting of an insertion of 6 nucleotides between residues 1012 and 1013, leading to the insertion of arginine and lysine (aat agc cct caa aga aaa acg aga/gga encoding N-S-P-Q-R-K-T-R/G → aat agc cct caa a  ga aaa acg aga/gga encoding N-S-P-Q-R*-*K*-*R-K-T-R/G, Table 3). The insertion was also detected at a low frequency (<5%) in the inoculum. Selection of viruses encoding arginine and lysine insertion was highly favoured and rapidly selected *in vivo*, and the disease outcome of this selection replicated our findings in the original trial with M6 (Table 3). The duplication in the HACS motif was detected in brain tissue of all chickens inoculated by ONO (*n* = 6) and IV (*n* = 6) routes, constituting 98.4–99.3% and 98.3–98.9% of viral nucleic acid sequences, respectively. This suggests that initial replication in mucosal epithelia is not an absolute requirement for selection of viruses with expanded HACS motifs, and that the vascular system does not differentially favour mid-length sequences.

### The H5 LPAIV HACS sequence motif is highly stable, even in the presence of strong selection pressure

We have demonstrated that incorporation of a single basic amino acid into a LPAIV motif may drive motif extension and conversion to high pathogenicity. Next, we wanted to show if low pathogenic variants (those with only two, non-consecutive, basic amino acids in the HACS) would be as prone to sequence extension, given the optimal conditions for doing so. Our premise was that highly pathogenic variants replicate predominantly in the ideal environment of the vascular compartment; if the mucosa is a barrier to entry into the vascular compartment, then direct IV inoculation can evade this barrier and favour pathotype switching. Therefore, inoculation of virus directly into the blood would help to drive this selection; in addition, inoculation of larger numbers of chickens would also raise the likelihood of conversion. To this end, we inoculated chickens by the IV (*n* = 10) and ONO routes (*n* = 25) and observed them for infection and disease.

Clinical signs were similar for both the ONO and IV groups inoculated with rgLPAIV, and included diarrhoea, drooping of the head, ruffled feathers, sleepiness and cyanosis of the comb. Morbidity was evident in 32% (8 of 25) and 80% (8 of 10) chickens in the ONO and IV group, respectively, and all but one (#28, IV inoculation) presented with neurological signs, including head twitching, torticollis, ataxia and paralysis. One chicken (#28) from the IV group required euthanasia at 2 dpi with HPAI-like clinical signs, including severe depression and recumbency, but with no neurological signs. Sequence analysis of virus re-isolated from the brain of chicken #28 identified a H5 LPAIV HACS motif. The survival curves are shown in Fig. 4. The median time from challenge to the onset of disease, in those chickens that developed HPAI-like or neurological disease were 196.4 (range: 120.0–240.5) and 144.0 hpi (range 47.0–262.3) for ONO and IV inoculation routes, respectively (*t*-test, *P* = 0.3468). Additionally, the frequency of neurological disease presentation did not significantly differ between ONO and IV inoculation routes (Fisher’s exact test, *P* = 0.0619).

Tissue tropism of rgLPAIV infection was not influenced by the inoculation route (Supplementary Table S3). Viral antigen was found to occur mainly in the epithelium of the kidney, in association with tubular degeneration and mononuclear cell interstitial nephritis, and in the epithelium of the nasal gland and infra-orbital sinus. All ONO and IV rgLPAIV inoculated chickens that displayed neurological signs were found to have histopathological lesion(s), viral antigen, and/or infectious virus in brain tissue. Neurological signs and neural lesions were associated with mild perivascular cuffing and glial response. Viral antigen within the brain was restricted to neuron cell bodies and astrocytes. Virus replication within vascular endothelium was observed only in one chicken (#28), which was challenged by the IV route and euthanased at day 2 pi. In this bird, viral antigen was observed at low levels within capillary endothelium in heart tissue and dermis of the comb; viral antigen was also present in cardiomyocytes. However, sequence analysis of virus re-isolated from this bird and others in the trial indicated that the virus maintained a LPAIV HACS motif.

Next generation sequencing performed on oropharyngeal swabs (*n* = 4) or brain tissue (*n* = 4) extracted from rgLPAIV inoculated chickens revealed that the viral population maintained a LPAIV HACS motif following *in vivo* passage, indicating that the HACS motif is stable, even in the presence of strong selection pressure. A mutation flanking the HACS motif was detected in one chicken (#26,), resulting in a PQ_RETR/G → LQ_RETR/G substitution (1010 c > t single nucleotide polymorphism (SNP) detected in 1.1% of sequences), further indicating possible tolerance of aliphatic residues at P6^48^. NGS performed on brain tissue extracted from chickens that exhibited neurological signs revealed an enrichment of the 377 a > g/c (N126T/S) SNP in the neuraminidase gene. This SNP constituted 1.3% (377 a > c; N126T) and 4.1% (377 a > g; N126S) of viral species in the inocula, and was enriched up to 99.98% of viral sequences detected. Of the two amino acids encoded by the SNP at position N126T/S, the presence of Ser (63.7–99.9% of species; detected in 8/8 rgLPAIV NGS samples) seemed to confer a greater fitness advantage in chickens than Thr (7.2–22.9% of species, detected in 5/8 rgLPAIV NGS samples). This SNP results in the loss of a predicted *N*-glycosylation site, which has been previously associated with neurovirulence of influenza A virus in mice (A/WSN/1933) (Asn146, N2 numbering)^49^.

These results indicate that no reversion to a HPAI pathotype or a highly pathogenic HACS motif occurred in rgLPAIV inoculated chickens. Despite one rgLPAIV inoculated chicken displaying clinical and pathological signs consistent with HPAIV infection, a HACS motif consistent with H5 LPAIV was maintained. The H5N1 LPAIV sequence is evidently highly stable and confers a high fitness advantage within the A/Vietnam/1203 backbone.

## Discussion

In this study, we examined the molecular pathogenesis and evolution of H5N1 HPAIV HACS motif from low pathogenic precursor viruses. Our findings inform the evolution of H5 viruses, but to a lesser extent H7 influenza viruses – the other principle subtype that develops high pathogenicity in chickens – as the H7 cleavage site motif has significant differences in its motif structure. Utilising a reverse genetics approach and the A/Viet Nam/1203/2004 Eurasian clade 1 H5N1 HPAIV, we systematically generated recombinant viruses harbouring HACS motifs with differences in the number of basic residues, or mutations at select sites, and compared their infection dynamics in chickens. Although previous studies have investigated the influence of variations of the HACS motif on influenza pathogenicity^27,33–40,50,51^, our study comprehensively performed systematic mutagenesis of the H5 HACS motif to assess the influence of HACS length and composition on H5N1 pathogenicity, pathogenesis, viral fitness and evolution in chickens. Our findings indicate that in chickens, there is relatively minor selection pressure on monobasic (LPAIV) and extended pHACS (HPAIV) sequences, however, mid-length pHACS are rapidly replaced by extended forms. This not only implies that in chickens, viruses harbouring LPAIV or HPAIV HACS motifs exhibit high viral fitness, but also that viruses harbouring mid-length pHACS motifs exhibit low fitness and are of a transient nature. Similar selection pressure may apply to avian influenza viruses under natural conditions and may explain some of the variation and pathogenicity seen with field isolates.

Our results are consistent with the low frequency of mid-length HACS motifs and the higher frequency of monobasic LPAIV and extended HPAIV pHACS motifs found in nature^19^. LPAIVs are commonly isolated from domestic and wild waterbirds and also in domestic gallinaceous species^52^. They are adapted for mucosal replication and transmission through the faecal-oral route and, as they are generally innocuous, they are able to maintain a fitness advantage with minimal genetic change. In contrast, viruses with extended pHACS motifs replicate with maximal efficiency within the host’s parenchymal tissues, causing acute, severe disease. Mid-length HPAIV pHACS motifs are not preferred as selection favours extended sequences. Of interest is whether continued passage of mutant viruses would result in further expansion of the HACS motif to yield viruses that harbour seven basic amino acids in the HACS motif, which seems to represent a fitness peak for naturally occurring HPAIVs^19^.

We measured pathogenicity by clinical signs, time of onset of morbidity, and tropism for vascular endothelium. Pathogenicity for AIV is usually scored using a measure of chicken mortality^53^. For this study, we incorporated endotheliotropism as an additional marker to better reflect overall pathogenesis. Endotheliotropism is a marked feature of H5N1 HPAIV replication in gallinaceous species^18,22–25,27,28,54,55^ and is the principle determinant of pathogenicity. This predilection for replication in endothelial cells can be annulled by removal of the pHACS^27,33–36^, indicating that the pHACS motif is a major determinant of endothelial tropism. Our study revealed a clear correlation between pathogenicity and the ability of the virus to replicate in vascular endothelium. Incorporation of one additional basic residue into the LPAIV HACS, to form a mid-length pHACS motif, was sufficient for pathotype conversion, with appearance of high mortality and endothelial tropism.

Wild type LPAIVs do not usually replicate in vascular endothelium, but will typically replicate in epithelium, particularly of the respiratory tract and kidneys, and this type of infection leads to limited disease. Such infection was observed in chickens infected with rgLPAIV in this study, with antigen observed in the nasal glands and kidney epithelium of some birds. However, we also observed replication in brain, with manifestation of neurological signs. This is not typical of LPAIV infection, and emphasises that the rgLPAIV is an artificial construct that may not be fully representative of naturally occurring LPAIV forms. Its replication in brain and other parenchymal tissues indicates that the HA protein is able to be cleaved, albeit inefficiently, in tissues that are not thought to produce trypsin-like enzymes. Moreover the N126S/T neuraminidase mutation identified functions to sequester plasminogen, leading to cleavage of HA, as has been demonstrated for the equivalent position (N2 numbering: N146S/T) of A/WSN/1933 (H1N1)^56^. Our study also indicates that the virus is able to be spread via the circulation, despite the lack of evidence that it replicates within the vascular system.

As measures of within-host fitness, we determined the survival times of infected chickens, we characterised the amount of virus in tissues and we measured the relative frequencies of HACS type through gene sequencing. Rapid disease progression occurred for viruses with extended pHACS motifs, with longer survival times for those with shorter (mid-length) motifs. High viral loads, with minimal variation between challenge groups, were detected in visceral organs from chickens inoculated with highly pathogenic viruses. Comparisons between viral loads detected in swabs from the oropharynx, and to a lesser extent, the cloaca, revealed a slight positive correlation between the number of basic residues within the pHACS motif and quantity of virus in oropharyngeal and cloacal fluids. Increasing the number of basic residues within the HACS motif increases the stability of the substrate-protease interaction due to an increase in the number of intermolecular hydrogen bonds formed^57^. A fitness advantage is likely conferred as HA is stabilised in the furin substrate-binding domain, facilitating increased HA cleavage efficiency. This fitness advantage may be important in the context of a fulminant, peracute infection as the ability to shed increased virus loads in a relatively short timeframe is likely to increase the potential for transmission^58^. Transmission studies with each of the high pathogenicity viruses would be of interest to determine the influence on transmission dynamics and may explain why a large proportion of naturally occurring H5 HPAIV isolates harbour an extended pHACS motif.

Extension of the HACS motif to include additional basic residues facilitates cleavage by the ubiquitously expressed protease, furin^20^. Furin’s ability to cleave basic substrates is modulated by the presence of acidic, negatively charged, residues at S1, S2 and S4 within the substrate binding domain^59^. The presence of additional basic residues, which contribute to an increased positive charge in the HACS motif, facilitates interactions with the negatively charged (acidic) residues within furin’s substrate binding domain. Increasing the number of basic residues in the HACS motif likely stabilises the interaction between furin and HA, leading to increased cleavage efficiency. Moreover, the preference for an expanded HPAIV pHACS motif may aid tolerance of rare mutations that introduce non-basic residues into the HACS motif during genome replication. Experimental evidence herein suggests that mutations at key sites in the H5 HACS motif (P4, M1; P2, M2) were tolerated *in vivo*, despite having a profound detrimental effect on cleavage activation *in vitro*^21,37,39^. The presence of multiple basic residues flanking these residues may compensate for sub-optimal interactions at these key sites. However, this may be highly dependent on the subtype and molecular sequence of the HACS motif, as mutation of P4 in the H7 HACS motif has been demonstrated to modulate pathogenicity^50^.

The VN1203 backbone used in this study may have contributed to pathotype conversion. The polygenic nature of AIV pathogenicity is well documented^33,60,61^, and VN1203 already possesses the optimal genetic constellation for acquisition of pathogenicity. Previously, incorporation of a pHACS motif into a LPAIV H3N8 backbone did not facilitate pathotype conversion^61^. Although H3 AIVs have not been demonstrated to support a highly pathogenic pathotype, other low-pathogenicity HA subtypes have, including H2, H4, H8 and H14^62^. Additional HACS motif mutagenesis studies utilising H5N1 isolates with various genetic backbones may shed light on whether pathotype conversion occurs as rapidly. This information could lead to the identification of optimal genetic constellations for subsequent acquisition of high pathogenicity.

Currently, despite the AIV HACS motif being extensively studied, the mechanisms and selection pressures that lead to expansion of the HACS motif in chickens is still unclear. In the present study, mutation events in the HACS motif appeared to occur in the chicken embryo in which the inocula were generated. Most of the mutations observed were substitutions or insertions of nucleotides encoding for basic amino acids. We hypothesise that the mutations observed in the HACS motif are selected within passage in chickens to optimise the interaction of the pHACS with furin.

A duplication event was detected in the HACS motif of M6 (PQ_RKTR↓G → PQ_*RK*RKTR↓G). A similar duplication event in the HACS motif was observed during the 1994 Mexico HPAIV outbreak^63,64^. Also, an insertion of a basic amino acid residue (arginine) was detected in the HACS motif of M7. Polymerase slippage as a consequence of HA RNA stem-loop secondary structure has been postulated to facilitating the incorporation of additional basic residues into the H5 HACS motif^64–66^. The mechanism for polymerase slippage is yet to be confirmed unequivocally, and as such, viruses that harbour mid-length HACS motifs provide a unique opportunity to dissect and confirm the molecular mechanism leading to insertion of additional basic residues in the HACS motif. We also observed substitutions (M1, M5) and these appear to have arisen from simple substitution of single bases within the codons.

The stability of the short LP motif suggests that it has high fitness in chickens. It is not easily replaced by longer motifs, despite experimental procedures, such as IV inoculation, to help incite such events. Therefore, evolution of high pathogenicity is not a common event and would require specific conditions, such as high host density, to enhance the selection of the initial mid-length HACS forms. But once these mid-length forms are generated, this may lead to escalating selection for extended motifs and the rapid emergence of highly pathogenic variants.

In summary, we have characterised the relative fitness of mutations that arise during H5 avian influenza virus evolution. Our study highlights that the critical bottleneck in evolution of HPAIVs lies in the switch of LP motifs to incorporate a single additional basic amino acid. Upon overcoming this evolutionary constraint, expansion of mid-length HACS motifs towards extended forms occurs rapidly. Identification of the molecular mechanism driving expansion of the HACS motif would not only lead to a greater understanding of the biology of influenza viruses, it would underpin our understanding of the mechanisms leading to the emergence and evolution of HPAIVs from low pathogenic precursors.

## Materials and Methods

### Cells

Madin Darby canine kidney (MDCK) (ATCC CCL-34) and Vero (ATCC CCL-81) cells were cultured in Minimal Essential Medium (MEM), and human embryonic kidney 293 T (HEK293T) cells (ATCC CCL-3216) were cultured in Dulbecco’s modified Eagle medium (DMEM). Media was supplemented to a final concentration of 10% (v/v) foetal calf serum (FCS), 10 mM HEPES, 2 mM L-glutamine, 100 U/ml benzylpenicillin, 100 µg/ml streptomycin, 50 µg/ml gentamicin and 2.5 µg/ml amphotericin B. Cell cultures were maintained at 37 °C with 5% CO_2_.

### Generation of viruses by reverse genetics

All reverse genetics viruses used in this study were derived from H5N1 HPAIV, A/Viet Nam/1203/2004. Mutations were introduced into the pHACS motif using QuikChange II-E site-directed mutagenesis kit (Stratagene) according to the manufacturer’s instructions. Mutagenesis oligo sequences are available upon request. Reverse genetics rescue of engineered viruses was performed as previously described^67^. Briefly, co-cultures of MDCK and HEK293T cells were transfected with the VN1203 eight-plasmid reverse genetics system using FuGene 6 (Roche). Transfection media was aspirated 6 hrs post-transfection, prior to the addition of Opti-MEM media containing 1 µg/ml TPCK-treated trypsin. Tissue culture supernatant (TCSN) was harvested 96 hrs post-transfection, clarified by centrifugation and stored at −80 °C.

Virus stocks were generated by passage of reverse genetics TCSN in the allantoic cavity of 10-day-old SPF embryonated chicken eggs. Infectious allantoic fluid was harvested, clarified by centrifugation and stored at −80 °C. The HA gene sequence was verified by Sanger sequencing prior to inoculation of chickens. The deduced amino acid sequences of engineered reverse genetics viruses are outlined in Table 1.

### Homology structure modelling of engineered HA glycoproteins

Protein homology structure modelling was performed with Swiss-Model (http://swissmodel.expasy.org/)^41–43^, based on the crystal structure of uncleaved influenza A HA_0_, PDB: 1HA0^44^. Predicted model structures were visualised and manipulated with Discovery Studio, version 4.1.0.14169 (BIOVIA).

### Animals and ethics statement

All animal experimental protocols were examined and approved by the CSIRO AAHL and Deakin University animal ethics committees, and were conducted in accordance with the Australian code for the care and use of animals for scientific purposes (Australian National Health and Medical Research Council). All embryonated chicken eggs used for virus growth and quantification were 10 days old at the time of inoculation.

All animal experimentation was performed in biosafety level 3 (BSL3) facilities, using the appropriate personal protective equipment, with powered air purifying respirators.

SPF white Leghorn embryonated eggs (Australian SPF Services) were incubated in a humidified incubator at 37 °C until hatch and subsequently housed in positive-pressure isolators. Upon reaching desired age, chickens were transferred to the BSL3 animal facility for acclimatisation, randomly assigned to groups and pre-inoculation samples taken. Challenge groups were housed separately. Food and water were available to chickens *ad libitum*.

### Study design

#### Initial pathogenesis study of HACS motif mutants

Four to six-week old SPF white Leghorn chickens were inoculated with ≥2 × 10^6^ TCID_50_ infectious allantoic fluid in sterile phosphate buffered saline (PBS) by the ONO route (*n* = 6 birds per group, with the exception of mutants M1, *n* = 4 and M2, *n* = 5). Negative controls were administered PBS diluent. Virus inocula were back-titrated to confirm the actual dose. Chickens were euthanased upon reaching a pre-defined clinical endpoint (moderate disease signs including infrequent eating and drinking, fluffed feathers, diarrhoea, sleepiness, depression and huddling) or if, for welfare reasons, a chicken was the sole occupant of the room. Pathogenicity was assessed by various methods, including percent morbidity and mortality, times to disease, viral loads in tissues, cell tropism, and tissue antigen amounts.

#### Follow-up studies with M6 and rgLPAIV

Four-week-old SPF white Leghorn chickens were inoculated with ≥2 × 10^6^ TCID_50_ mutant M6 by the ONO or IV route (*n* = 6 per group). Additionally, SPF chickens were inoculated with ≥2 × 10^6^ TCID_50_ rgLPAIV by the ONO or IV route (*n* = 25 and 10, respectively). Viral doses were confirmed by back titration of the inocula.

### Virus titration

Viral loads were quantified by median tissue culture infectious dose (TCID_50_) on Vero or MDCK cells in the presence of 1 µg/ml TPCK-treated trypsin. Tissue homogenates (~10%) or neat swab media (PBS containing 10 µg/ml bovine serum albumin, 500 U/ml benzylpenicillin, 500 µg/ml streptomycin and 50 µg/ml gentamicin) were serially diluted 10-fold and applied in quadruplicate to cell monolayers. Cytopathic effect was assessed five days post infection. Viral titres were calculated based on the Spearman-Karber method^68^. Additionally, the presence of infectious virus was confirmed by allantoic inoculation of triplicate 10-day-old embryonated chicken eggs with 100–200 µl of neat swab media or 10% tissue homogenate.

### RNA extraction, cDNA synthesis, Sanger sequencing and next generation sequencing

RNA was directly extracted from infected allantoic fluid, brain and/or lung tissue using RNeasy mini kit (QIAGEN) according to the manufacturer’s instructions. cDNA was synthesised using SuperScript III First-Strand synthesis system (Life Technologies) according to manufacturer’s instructions. AAHL in-house gene-specific primers were used to amplify the HA gene segment using high fidelity enzyme. Sanger sequencing was performed directly on PCR amplicons.

For NGS, total RNA was extracted from stock allantoic fluids or brain tissue (M6 and rgLPAIV) or oropharyngeal swab (rgLPAIV) using MagMAX Total Nucleic Acid Isolation Kit (Thermo Fisher Scientific) and cDNA was synthesised as described above. Partial amplification of mutant M6 HA gene (spanning the HACS motif) was performed using high fidelity enzyme (Kapa Biosystems) with HA specific primers (available upon request). Amplicons were visualised by DNA gel electrophoresis prior to purification. Multiplexing indices and sequencing adaptors were incorporated by a second round of PCR (Nextera XT Index Kit, Illumina). M6 amplicon libraries were purified (Ampure XP beads, Beckman Coulter), quantified, normalised and pooled prior to multiplex sequencing using 300-bp paired reads on Illumina MiSeq. rgLPAIV whole genome were amplified using high fidelity enzyme (Kapa Biosystems) using whole genome primers^69^. Whole genome libraries were tagmented, and multiplexing indices and sequencing adaptors were incorporated in a subsequent round of PCR using Nextera XT DNA Sample Preparation Kit (Illumina). Whole genome libraries of rgLPAIV were purified (Ampure XP beads, Beckman Coulter), quantified, normalised, pooled and subjected to multiplex sequencing using 250-bp paired reads on Illumina MiSeq. *In silico* NGS data analysis was performed as previously outlined^70^, with modifications. Whole genome sequencing (WGS) read mapping was modified as follows: mismatch, two; insertion/deletion, three. WGS variant filtering was modified as follows: independent counts, >10; minimum coverage: >999; average quality variant, >Q30; variant frequency: >1%. Amplicon read trimming were modified as follows: remove reads <200 bases. Amplicon variant filtering was modified as follows: independent counts, >two; average quality variant, >Q30; variant frequency, >1%.

### Histopathology and immunohistochemistry

Tissue samples harvested at necropsy were placed in 10% neutral buffered formalin (Australian Biostain). Preserved tissue samples were processed according to routine histological methods before embedding in paraffin wax and sectioning into 4 µm-thick sections.

A rabbit anti-*Influenzavirus A* nucleoprotein hyperimmune serum (generated in-house by AAHL Bioassay R&D Team) was used as the primary antibody to detect viral antigen in formalin-fixed paraffin embedded tissues. Tissue sections were deparaffinized before high pH antigen retrieval (Dako, Agilent Technologies) at 97 °C for 30 min. Endogenous peroxidases were blocked with 3% hydrogen peroxide for 10 min. Tissue sections were incubated with a 1:2,000 dilution of the primary antibody for 60 min, washed with TBS prior to a 20 min incubation with anti-rabbit horseradish peroxidase-conjugated secondary antibody (Dako, Agilent Technologies). Viral antigen was visualized by the addition of AEC substrate chromogen (Dako, Agilent Technologies) for 10 min, washed with TBS and counterstaining with Mayer’s haematoxylin before visualising by light microscopy.

The presence of influenza nucleoprotein antigen in tissues was assessed by light microscopy. Antigen quantities were scored according to a four-point scoring system: 0, no antigen; 1, rare antigen; 2, moderate antigen; 3, abundant antigen.

### Statistical analysis

Statistical analyses were performed using Prism 7, version 7.03 (GraphPad, La Jolla, USA). Unless stated otherwise, multiple comparisons were performed using a non-parametric Kruskal-Wallis test with a post hoc Dunn’s multiple comparisons test. Comparisons of survival curves was assessed with Log-rank (Mantel-Cox) test with Bonferroni-adjusted thresholds due to multiple comparisons. Error bars represent mean ± 95% confidence intervals (CI). Significance levels for are expressed as: *P < 0.05; **P < 0.01; ***P < 0.001.

### Data availability

All data generated or analysed during this study are included in this published article (and its Supplementary Information files).

## Electronic supplementary material

Supplementary information
