# Supplementary material for: Evolution of high pathogenicity of H5 avian influenza virus: haemagglutinin cleavage site selection of reverse-genetics mutants during passage in chickens
Source: Sci Rep. 2018 Aug 1;8:11518. doi: 10.1038/s41598-018-29944-z (PMC6070550; doi:10.1038/s41598-018-29944-z)
Supplement: Supplementary file 1 — Supplementary information [file 41598_2018_29944_MOESM1_ESM.pdf]

Supplementary Information

Evolution of high pathogenicity of H5 avian influenza virus: haemagglutinin cleavage site selection of reverse-genetics mutants during passage in chickens

Jasmina M. Luczo[1, 2, #a], Mary Tachedjian[1], Jennifer A. Harper[1], Jean S. Payne[1], Jeffrey M. Butler[1], Sandra I. Sapats[1], Suzanne L. Lowther[1], Wojtek P. Michalski[1], John Stambas[2], John Bingham[1]*

[1]Australian Animal Health Laboratory (AAHL), Commonwealth Scientific and Industrial Research Organisation (CSIRO), Geelong, Victoria, Australia

[2]School of Medicine, Deakin University, Geelong, Victoria, Australia

[#a]Current address: Center for Vaccines and Immunology, University of Georgia, Athens, Georgia, United States of America

Supplementary Table S1: Predicted furin cleavage scores within engineered HACS motifs.

Virus	HACS motif	Number of predicted sites[1]	Furin cleavage motif	Score
rgHPAIV	NSPQRERRRKKR/G	2	SPQRERR/RK	0.616
			ERRRKKR/GL	0.829
rgLPAIV	NSPQRE----TR/G	0	SPQRETR/GL	0.485
M5	NSPQRE----KR/G	1	SPQREKR/GL	0.787
M6	NSPQR----KTR/G	1	SPQRKTR/GL	0.572
M7	NSPQ----RKKR/G	1	SPQRKKR/GL	0.833
M4	NSPQRE--RKKR/G	1	QRERKKR/GL	0.803
M8	NSPQ---RRKKR/G	1	PQRRKKR/GL	0.758
M9	NSPQRE-RRKKR/G	2	SPQRERR/KK	0.556
			RERRKKR/GL	0.806
M2	NSPQRERRRKTR/G	2	SPQRERR/RK	0.628
			ERRRKTR/GL	0.584
M1	NSPQRERRQKKR/G	1	SPQRERR/QK	0.687
M3	NVPQRERRRKKR/G	2	VPQRERR/RK	0.547
			ERRRKKR/GL	0.829

[1] Furin cleavage site predictions performed on deduced amino acid sequences of HACS motifs using ProP 1.0 (http://www.cbs.dtu.dk/services/ProP/). Scores >0.5 predict cleavage by furin; higher scores reflect more confident predictions of cleavage efficiency.

Supplementary Table S2: Viral antigen detected in selected tissues of chickens following inoculation with reverse genetics viruses.

Challenge virus	Chicken identity	Euthanasia (dpi) [1]	Brain	Heart	Spleen	Lung	Kidney	Jejunum	Head[2]
rgLPAIV	77	14§	-	-	-	-	-	+	-
	79	14§	+	-	+	-	- ∂	-	+ (e)
	82	14§	-	-	-	-	-	-	-
	83	14§	- *	-	-	-	-	-	+ (e)
	87	14§	+	-	-	-	- ∂	-	+ (e)
	93	7 (n)	++	+	-	+	+	-	+ (e, ng)
rgHPAIV	81	1	+++	+++	+++	+++	n/s	n/s	n/s
	85	1	+++	+++	+++	+++	n/s	n/s	n/s
	88	1	+++	+++	+++	+++	+++	+++	+++
	89	1	+++	+++	+++	+++	+++	+++	+++
	90	1	+++	+++	+++	+++	+++	+++	+++
	92	1	+++	+++	+++	+++	+++	+++	+++
M5	7	2§	++	++	++	++	n/s	n/s	n/s
	8	1	+++	+++	+++	+++	n/s	n/s	n/s
	9	2 ⌂	+++	+++	++	++	n/s	n/s	n/s
	10	1	++	+++	++	+++	n/s	n/s	n/s
	11	1	+++	+++	++	+++	n/s	n/s	n/s
	12	1	++	++	+++	++	n/s	n/s	n/s
M6	13	1	+++	+++	++	+++	n/s	n/s	n/s
	14	1	+++	+++	+++	+++	n/s	n/s	n/s
	15	1	+++	+++	+++	+++	n/s	n/s	n/s
	16	1 (n)	+++	+++	+++	+++	n/s	n/s	n/s
	17	1	+++	+++	+++	+++	n/s	n/s	n/s
	18	1	+++	+++	+++	+++	n/s	n/s	n/s

[1] (n), neurological involvement; ⌂ found dead; § clinically healthy at euthanasia.
[2] (e), epidermis; (ng), nasal gland.
-, negative; +, rare; ++, common; +++ abundant antigen; n/s, not scored.
* brain lesions present in the absence of antigen; ∂ kidney lesion present in the absence of antigen.

Challenge virus	Chicken identity	Euthanasia (dpi) [1]	Brain	Heart	Spleen	Lung	Kidney	Jejunum	Head[2]
M7	27	2	+++	+++	+++	+++	n/s	n/s	n/s
	28	2 ⌂	+++	+++	+++	+++	n/s	n/s	n/s
	29	1	+++	+++	+++	+++	n/s	n/s	n/s
	30	1	++	+++	+++	+++	n/s	n/s	n/s
	31	1	+++	+++	+++	+++	n/s	n/s	n/s
	32	1	+++	+++	+++	+++	n/s	n/s	n/s
M4	53	1	++	+++	+++	+++	++	n/s	+++
	54	1	+++	+++	+++	+++	+++	+++	+++
	55	1	++	+++	+++	+++	+++	n/s	++
	59	1	+++	+++	+++	+++	+++	+++	+++
	61	1	++	+++	+++	+++	+++	+++	+++
	74	1	+++	+++	+++	+++	+++	+++	++
M8	19	1	+++	+++	+++	+++	n/s	n/s	n/s
	20	1	+++	+++	+++	+++	n/s	n/s	n/s
	21	1	+++	+++	+++	+++	n/s	n/s	n/s
	45	1	+++	+++	+++	++	n/s	n/s	n/s
	46	1§	++	++	++	++	n/s	n/s	n/s
	47	1	+++	+++	+++	+++	n/s	n/s	n/s
M9	21	1	+++	+++	+++	+++	n/s	n/s	n/s
	22	1	+++	+++	+++	+++	n/s	n/s	n/s
	23	1	+++	+++	+++	+++	n/s	n/s	n/s
	24	1	+++	+++	+++	+++	n/s	n/s	n/s
	25	1	+++	+++	+++	+++	n/s	n/s	n/s
	26	1	+++	+++	+++	+++	n/s	n/s	n/s
M2	8	1	+++	+++	+++	+++	+++	+++	+++
	13	1	+++	+++	+++	+++	+++	+++	+++
	23	1	+++	+++	+++	+++	+++	+++	+++
	25	1	+++	+++	+++	+++	+++	+++	+++
	40	1	+++	+++	+++	+++	+++	+++	+++
M3	51	1	+++	+++	+++	+++	+++	n/s	+++

Challenge virus	Chicken identity	Euthanasia (dpi) [1]	Brain	Heart	Spleen	Lung	Kidney	Jejunum	Head[2]
M3 continued	52	1	++	+++	+++	++	+++	n/s	++
	57	1	+++	+++	+++	+++	+++	n/s	n/s
	58	1	+++	+++	+++	+++	+++	n/s	+++
	60	1	++	+++	++	++	++	n/s	++
	73	1	++	+++	++	++	++	n/s	n/s
M1	9	1	+++	+++	+++	+++	n/s	n/s	+++
	10	1[§]	+	++	++	+++	n/s	n/s	++
	15	1	+++	+++	+++	+++	n/s	+++	+++
	16	1	+++	+++	+++	+++	n/s	n/s	n/s

Supplementary Table S3: Viral antigen detected in selected tissues following oronasal-ocular or intravenous inoculation with rgLPAIV or mutant M6 (second study).

Challenge virus	Chicken identity	Euthanasia (dpi) [3]	Brain[4]	Heart	Spleen	Lung	Kidney	Head[5]
rgLPAIV ONO	1	6 (n)	+++ (ne)	-	-	-	++	+ (e) ++ (ng)
	2	14§	-	-	-	-	nt	-
	3	10 (n)	-*	-	-	-	- ∂	++ (ie)
	4	14§	-	-	-	-	++	-
	5	14§	-	-	-	-	-	-
	6	10 (n)	+ (ne)	+	-	-	-	+ (e)
	7	6 (n)	+++ (ne)	-	-	-	+	+ (e) ++ (ng)
	8	14§	-	-	-	-	- ∂	-
	9	14§	-	-	-	-	-	-
	10	8 (n)	++ (ne)	-	-	-	-	+ (e)
	11	14§	-	-	-	-	-	-
	12	8 (n)	+ (ne)	-	-	-	+ ∂	+
	13	14§	-	-	-	-	-	-
	14	14§	-	-	-	-	++	+ (e)
	15	14§	-	-	-	-	++	-
	16	14§	-	-	-	-	-	-
	17	14§	-	-	-	-	+	-
	18	14§	-	-	-	-	-	-
	19	14§	-	-	-	-	-	-

[3] (n), neurological signs; ⌂ found dead; § clinically healthy at euthanasia; Ω displayed HPAI-like clinical signs at euthanasia.
[4] (ne) neuronal staining
[5] (e), epidermis; (ng), nasal gland, (ie), inner ear, (ec), endothelial cell
-, negative; +, rare; ++, common; +++ abundant antigen; n/s, not scored.
* brain lesions present in the absence of antigen; ∂ kidney lesion present in the absence of antigen.
nt – no tissue sample

Challenge virus	Chicken identity	Euthanasia (dpi) [3]	Brain[4]	Heart	Spleen	Lung	Kidney	Head[5]
rgLPAIV ONO	20	9 (n)	++ (ne)	-	-	-	+	+ (ng) ++ (e)
	21	14§	-*	-	-	-	-	-
	22	14§	-	-	-	-	-	-
	23	14§	-	-	-	-	-	-
	24	14§	-	-	-	-	-	-
	25	8 (n)	++ (ne)	-	-	-	-	+
rgLPAIV IV	26	6 (n)	++ (ne)	-	-	-	+	+ (e)
	27	6 (n)	+++ (ne)	-	-	-	+	+
	28	2 Ω	+	+	+	+	+	+ (ec)
	29	14§	-*	-	-	-	-	+ (e)
	30	6 (n)	++ (ne)	-	-	-	+	+ (e) + (ng)
	31	14§	-	-	-	-	-	+ (e)
	32	6 (n)	++ (ne)	-	-	-	++	+ (e) + (ng)
	33	11 (n)	-*	-	-	-	-	+ (ng)
	34	11 (n)	+ (ne)	-	nt	-	-	-
	35	6 (n)	++ (n)e)	-	-	-	++	+ (e) + (ng)
M6 ONO	36	1	+++	+++	+++	+++	n/s	+++
	37	1	+++	+++	+++	+++	n/s	+++
	38	1	++	++	+++	+++	+++	+++
	39	1	++	+++	+++	+++	n/s	n/s
	40	1	++	++	+++	+++	n/s	n/s
	41	1	+++	+++	+++	+++	n/s	n/s
M6 IV	42	<1	+++	+++	+++	+++	n/s	n/s
	43	<1	+++	+++	+++	+++	n/s	n/s
	44	<1	+++	+++	+++	+++	n/s	n/s
	45	<1 ⌂	+++	+++	+++	+++	n/s	n/s
	46	<1	+++	+++	+++	+++	n/s	n/s
	47	<1	+++	+++	+++	+++	n/s	n/s

Supplementary Figure S1: Viral shedding 24 h post-inoculation in chickens with highly pathogenic avian influenza viruses.

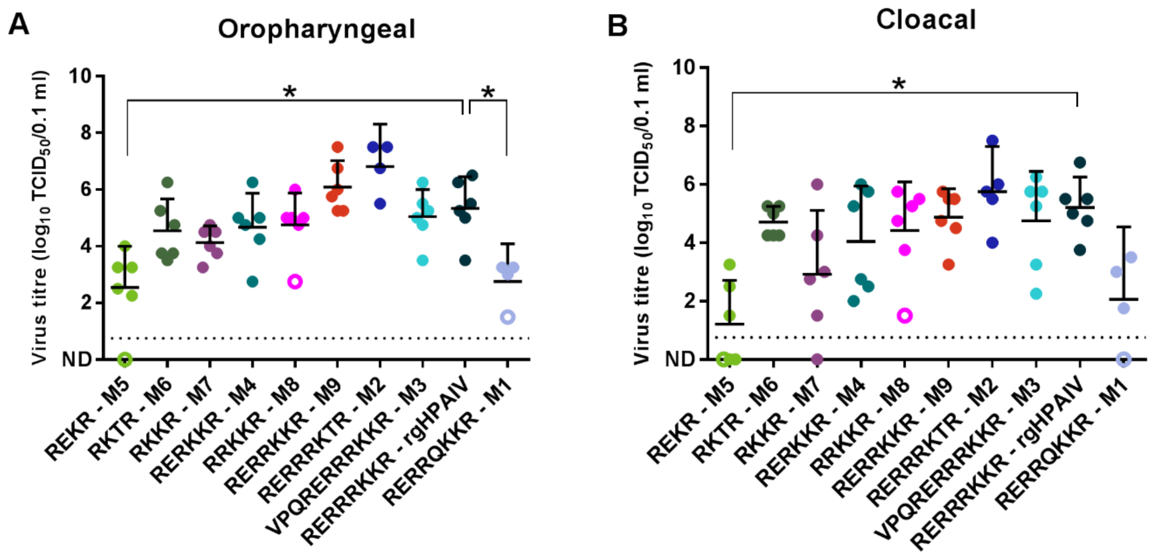

Viral loads in oropharyngeal (A) and cloacal (B) swabs were determined by titration on Vero cells. Viral loads in chickens that lacked clinical disease signs at euthanasia are indicated by open circles. Lower limit of assay detection indicated by dotted line. ND, not detected. Error bars represent mean ±95% CI. *n* = 4-6. **P* = < 0.05.

Supplementary Figure S2: Viral loads in tissues following oronasal-ocular inoculation of chickens with highly pathogenic viruses.

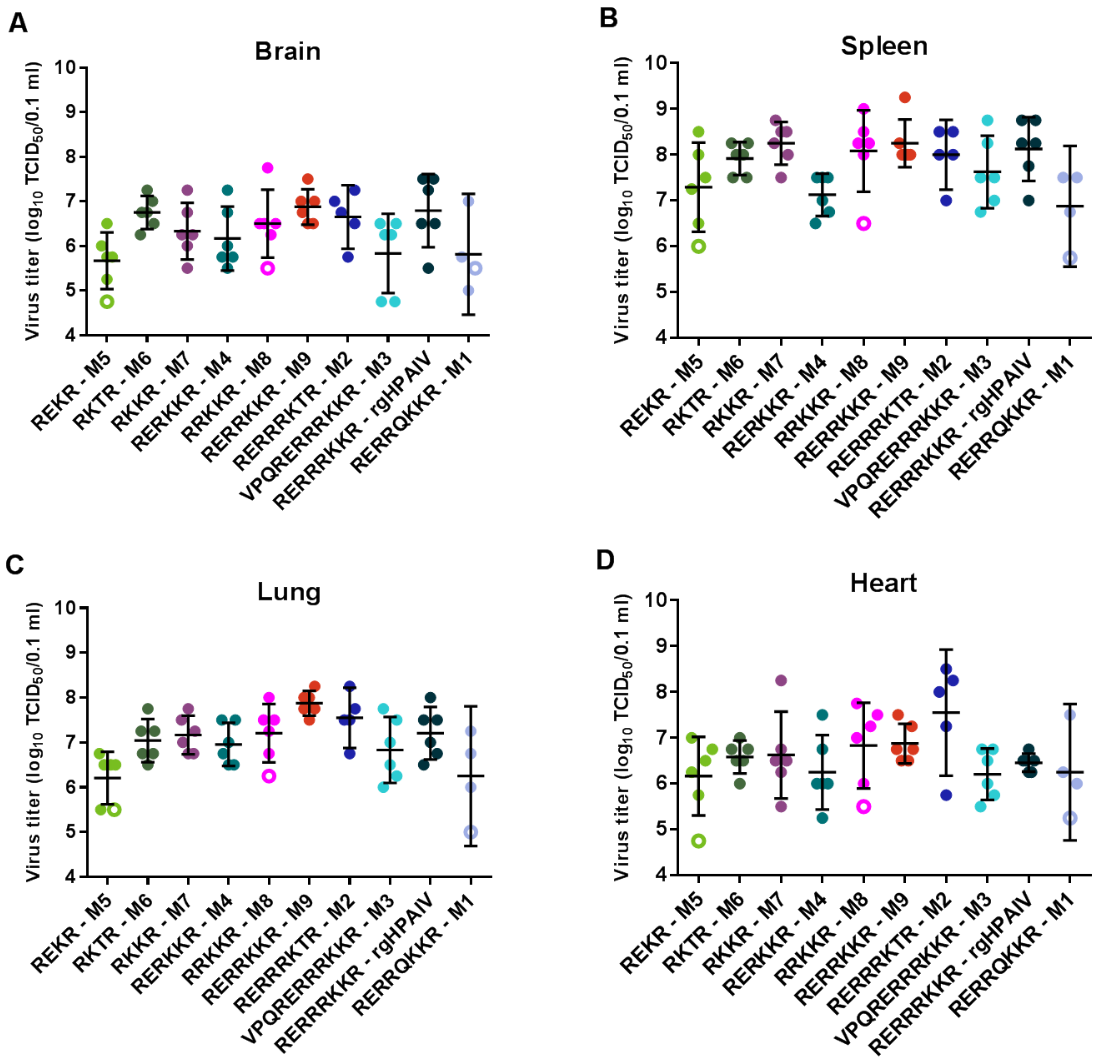

Viral loads in brain (A), spleen (B), lung (C) and heart (D) were determined by virus titration on Vero cells. Viral loads in chickens that did not display clinical disease signs are indicated by open circles. Comparisons of viral loads in rgHPAIV and engineered virus infected tissues returned a non-significant result (Kruskal-Wallis test). *n* = 4-6. Error bars represent mean ±95% CI.

Supplementary Figure S3: Viral antigen in rgLPAIV infected chicken exhibiting neurological signs.

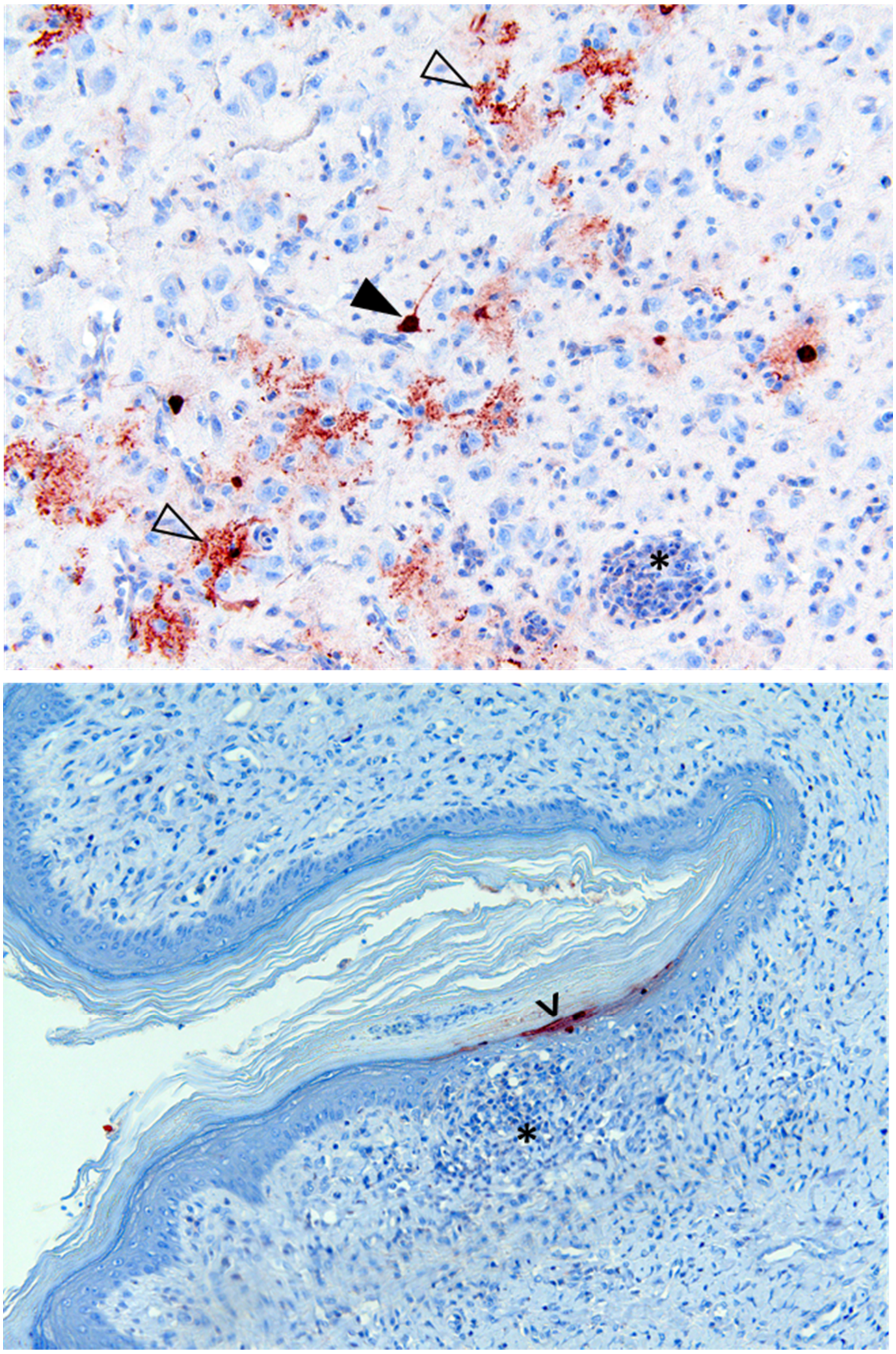

Influenzavirus A nucleoprotein antigen (brown) by immunohistochemistry in brain (top) and skin of comb (bottom) of rgLPAIV infected chicken exhibiting neurological signs (#93). Viral antigen is present in neurons (filled arrowhead), astrocytes (unfilled arrowhead) and epidermis (>). Inflammatory reactions are evident in both tissues (*).
